# Near Infrared Spectroscopy Enables Differentiation of Mechanically and Enzymatically Induced Cartilage Injuries

**DOI:** 10.1007/s10439-020-02506-z

**Published:** 2020-04-16

**Authors:** Ervin Nippolainen, Rubina Shaikh, Vesa Virtanen, Lassi Rieppo, Simo Saarakkala, Juha Töyräs, Isaac O. Afara

**Affiliations:** 1 Department of Applied Physics, University of Eastern Finland, P.O. Box 1627, 70211 Kuopio, Finland; 2 Diagnostic Imaging Center, Kuopio University Hospital, Kuopio, Finland; 3 Research Unit of Medical Imaging, Physics and Technology, Faculty of Medicine, University of Oulu, Oulu, Finland; 4 Department of Diagnostic Radiology, Oulu University Hospital, Oulu, Finland; 5 School of Information Technology and Electrical Engineering, The University of Queensland, Brisbane, Australia

**Keywords:** Articular cartilage, Post-traumatic osteoarthritis, NIR spectroscopy, Cartilage damage, Biomechanics

## Abstract

This study evaluates the feasibility of near infrared (NIR) spectroscopy to distinguish between different cartilage injury types associated with post-traumatic osteoarthritis and idiopathic osteoarthritis (OA) induced by mechanical and enzymatic damages. Bovine osteochondral samples (*n *= 72) were subjected to mechanical (*n *= 24) and enzymatic (*n *= 36) damage; NIR spectral measurements were acquired from each sample before and after damage, and from a separate control group (*n *= 12). Biomechanical measurements were then conducted to determine the functional integrity of the samples. NIR spectral variations resulting from different damage types were investigated and the samples classified using partial least squares discriminant analysis (PLS-DA). Partial least squares regression (PLSR) was then employed to investigate the relationship between the NIR spectra and biomechanical properties of the samples. Results of the study demonstrate that substantial spectral changes occur in the region of 1700–2200 nm due to tissue damages, while differences between enzymatically and mechanically induced damages can be observed mainly in the region of 1780–1810 nm. We conclude that NIR spectroscopy, combined with multivariate analysis, is capable of discriminating between cartilage injuries that mimic idiopathic OA and traumatic injuries based on specific spectral features. This information could be useful in determining the optimal treatment strategy during cartilage repair in arthroscopy.

## Introduction

Osteoarthritis (OA) is a complex and multifaceted disease of articulating joints. The condition is mainly characterized by severe pain, restricted joint movement and erosion of articular cartilage, the layered tissue covering the ends of bones in articulating joints. This highly specialized, avascular and aneural tissue is responsible for load transmission and lubrication in the joint. Cartilage is comprised primarily of a framework of collagen fibers and entrapped proteoglycan macromolecules, the extracellular matrix (ECM) components of the tissue (20–35%), and water (65–80%).^36^ Alteration of the collagen network^12^ or loss of proteoglycan macromolecules^14^ are often indicative signs of articular cartilage degeneration. Although OA could be a result of aging, post-traumatic osteoarthritis (PTOA), a common form of the disease, could be initiated by mechanical overloading of the joint, resulting in an injury to articular cartilage.^9^,^11^,^20^ Early diagnosis of cartilage injuries is important for prevention of progression of PTOA, either *via* surgical or pharmacological interventions.^6^

Cartilage injuries differ in extent and type of tissue damage and the response of the tissue to the injury.^11^–^13^ While joint degeneration could be associated with alteration of matrix macromolecular framework and cells, without any mechanical disruption of the tissue, it can also initiate from fracture or rupture of the cartilage matrix causing visible splits in the articular surface. In order to study the pathological processes involved in cartilage degeneration and to test potential treatment options, numerous *in vitro* and *in vivo* injury models have been used,^24^,^31^,^42^,^43^ as well as various types of enzymes, to simulate the degradation of ECM. Enzymatic degradation tends to result in gradual loss of PG from the superficial to deep cartilage, mimicking symptoms of idiopathic OA.^1^ Enzymatic treatment allows targeted and controlled degradation of the tissue. Moody *et al*.^24^ examined the consistency of the outcome of artificial modification of cartilage using trypsin to induce proteoglycan depletion. Saarakkala *et al*.^31^ and Wang *et al*.^43^ used enzymatically digested bovine cartilage with purified collagenase and trypsin to evaluate the capacity of high-frequency ultrasound to detect spatial and temporal changes in matrix composition and structure. Wagner *et al*.^42^ demonstrated that collagenase-induced damage to cartilage can be visualized using high-resolution nuclear magnetic resonance microscopy. In order to simulate traumatic impact injuries, it is common practice in laboratories to apply excessive mechanical loads or stresses to articular cartilage explants in order to initiate mechanical damage.^4^,^15^,^16^,^21^ Injurious impact loading of the joint is commonly associated with PTOA. Although cartilage degradation pathophysiology is broadly similar to that of idiopathic primary OA, patients with PTOA often experience progressive joint degeneration.^11^

Cartilage damage can be assessed and repaired during arthroscopic surgery. However, traditional arthroscopic examination of cartilage injuries is subjective and has poor intra- and inter-observer reliability.^37^ Moreover, it merely shows visible alteration of the articular surface, which often occurs in combination with other joint injuries. It has been suggested that the effectiveness of arthroscopic examination could be radically improved by complimentary diagnostic methods such as near infrared (NIR) spectroscopy, optical coherent tomography (OCT), mechanical indentation, and ultrasound imaging, which are minimally invasive, non-destructive, sensitive, and objective.^27^,^30^,^31^,^34^

Optical spectroscopy is an effective, non-destructive method for determining the structure and composition of materials, including those of biological origins. In particular, NIR spectroscopy is among the most useful optical methods for qualitative and quantitative arthroscopic assessment and characterization of the properties of articular cartilage.^3^,^8^,^19^,^25^,^38^ NIR spectroscopy utilizes shorter wavelengths of the electromagnetic spectrum than mid-infrared spectroscopy and is based on excitation of overtone and combination vibrations, resulting in weak spectrum with broad and overlapping peaks.^25^,^36^ Nevertheless, NIR spectroscopy offers superior penetration depth into soft tissues compared to other optical spectroscopic techniques, allowing non-destructive full-depth evaluation of articular cartilage.^5^ Furthermore, NIR spectroscopy has been shown to be a versatile technique for arthroscopic evaluation of connective tissue integrity during surgery. This was first demonstrated by Spahn *et al*.^38^ for evaluation of human cartilage, and more recently with more accurate results by Sarin *et al*.^32^ and Prakash *et al*.^27^ using a prototype NIR fiber optic arthroscopic probe for estimating the integrity of equine and human cartilage, respectively. Problems associated with NIR spectroscopy, including overlapping spectral bands, can be overcome by using multivariate data analysis techniques, such as principal component analysis (PCA),^2^ partial least square regression (PLSR)^28^ and partial least squares discriminant analysis (PLS-DA), coupled with spectral preprocessing.

Although NIR spectroscopy has been shown to be a promising technique for *in vivo* evaluation of cartilage structure, composition and integrity,^32^ no study has evaluated its capacity to differentiate between trauma-related structural injury and osteoarthritis related changes in tissue composition. This information could be critical in determining the optimal treatment remedy during cartilage repair in arthroscopy. We hypothesize that NIR spectroscopy is sensitive to changes in matrix structure and biochemical composition associated with the different injury types (i.e., structural and no structural injuries with and without PG or collagen losses) and could provide a tool for characterizing the origin of various physiological degenerative changes in the cartilage matrix.

The aim of this study is to evaluate the capacity of NIR spectroscopy to distinguish between different cartilage injury types associated with PTOA and idiopathic OA induced by respective mechanical and enzymatic damages in bovine cartilage.

## Materials and Methods

### Sample Preparation

**Figure 1:**
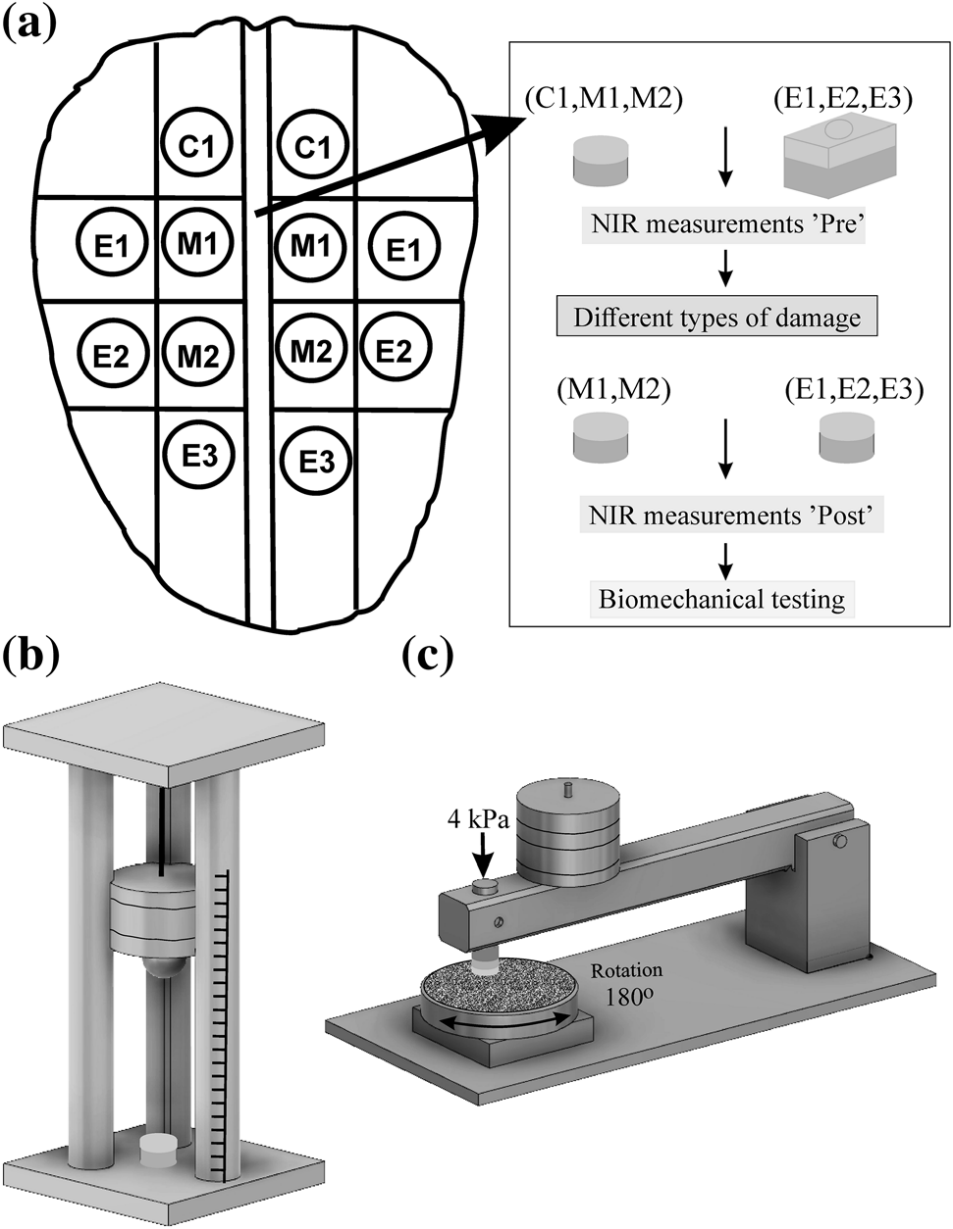
(a) Anatomical location of bovine osteochondral samples: C1-control, E1-collagenase 24 h, M1-impact, E2-collagenase 90 min, M2-abrasion, and E3-trypsin. (b) Custom-made drop-tower used to induce impact injury. (c) Custom-made grinding tool used to sample abrasion.

Patellae (*n* = 10) with visually normal cartilage surface were extracted from bovine (age 14–22 months) knee joints obtained from a local abattoir. Osteochondral samples (*n* = 72) were harvested by using biopsy punch (diameter = 7 mm) from different anatomical locations of medial and/or lateral sides of patellae (Fig. 1a). The samples were divided into groups based on tissue damage protocol as follows: intact control samples (*n *= 12), mechanical damage (*n *= 24), and enzymatic damage (*n *= 36). Cylindrical osteochondral plugs with 7 mm diameter were prepared for intact control and mechanical damage groups. In case of enzymatic damage, to avoid lateral penetration of the enzyme into the tissue, larger rectangular samples (10 × 15 mm) were enzymatically treated followed by extraction of 7 mm cylindrical plugs from the center of the degraded tissue.

### Mechanical Damage

The samples in this group were further divided into two subgroups. The first subgroup (M1, *n *= 12) was subjected to mechanical injury *via* impact loading using a custom-made drop tower (Fig. 1b) as described in Kokkonen *et al*.^21^ Briefly, a stainless-steel impactor (200 g) with a polished steel ball (*d *= 1 cm) at the end was dropped onto the sample from a height of 7.5 cm. The impactor dropping height, and thus energy delivered to the cartilage surface, was determined based on preliminary assessments to create minor chondral cracks on the cartilage surface.^21^ To prevent creep deformation, the impactor was lifted from the sample immediately after the impact.

The second subgroup (M2, *n *= 12) was subjected to mechanical abrasion surface damage using P80 sandpaper (corresponding to particle size of 200 *µ*m). Using a custom-made grinding tool (Fig. 1c), the surface of each sample was abraded under constant stress (4 kPa) by a rotating (180° rotation) metal plate with sandpaper glued on to it. The cartilage surfaces were abraded along two perpendicular directions. Following mechanical damage, the samples of both subgroups were immersed in phosphate buffered saline (PBS) solution and allowed to recover for 1 h.

### Enzymatic Degradation

Two enzymes were used for degradation of the samples in group 2. Collagenase D (Sigma Aldrich) was used for the degradation of the collagen network,^35^ while trypsin (T4299, Sigma Aldrich) was used for proteoglycan digestion (with minor collateral effect on the collagen network).^17^ The samples in group 2 were further divided into three subgroups according to the enzyme and duration of treatment as follows: collagenase 24 h (E1, *n *= 12), collagenase 90 min (E2, *n *= 12) and trypsin 30 min (E3, *n *= 12). The prewarmed samples were incubated at 37 °C and 5% CO_2_ in PBS solution containing the respective enzymes (0.1 mg/mL for Collagenase D, and 0.5 mg/mL for Trypsin) and supplemented with antibiotics, including Penicillin–Streptomycin–Amphotericin B (100 U/mL Penicillin, 100 *µ*g/mL Streptomycin and 0.25 *µ*g/mL Amphotericin B, stabilized, Sigma-Aldrich) for the different times specified previously. The incubation times of 30 min for trypsin and 90 min for collagenase were applied to induce mild cartilage degradation that mimic early OA. In order to induce severe damage, long incubation time of 24 h in collagenase was applied.

### NIR Spectroscopy

NIR spectral measurements of each sample were obtained before (Pre) and after (Post) degradation. NIR measurements (3 spectra per sample) were carried out before and after injury at the center of samples immersed in PBS mimicking *in vivo* arthroscopy. Each spectral measurement is the average of 50 acquisitions, with integration time of 16 ms. The NIR system consisted of a spectrometer (AvaSpecULS2048XL, Avantes BW, grating 75 lines/mm, slit 50 *µ*m which give in λ = 1.0–2.5 *µ*m resolution = 6.4 nm), light source (Avalight-HAL-S, Avantes BW, Netherlands) and a custom diffuse reflectance arthroscopic fiber optic probe.^29^,^33^ The probe tip was perpendicular to and in contact with the sample surface during the measurements. The tip of the reusable stainless-steel fiber probe (*d *= 3.25 mm) resembles the shape of a traditional arthroscopic hook. The probe tip window (*d *= 2 mm) contains 114 optical fibers (*d *= 100 *µ*m), with 100 fibers emitting and 14 fibers (7 + 7) collecting light to the spectrometers. Avasoft software (version 8.7.0, Avantes BV) was used for spectral data acquisition.^32^

### Spectral Preprocessing

Spectral preprocessing was conducted using *nippy* (https://github.com/uef-bbc/nippy), an open source spectral preprocessing toolbox.^40^ The toolbox contains multiple preprocessing methods and can be extended with custom functions. Preprocessing methods from five categories, i.e., clipping, scatter correction, smoothing, derivation, and trimming, were implemented. The best model was obtained with the combination of 1st order derivative and Savitzky–Golay filtering (filter length 5 nm) with standard normal variate (SNV) scatter correction. Spectra were interpolated in 1000–2200 nm range for further data analysis.

### Multivariate Data Analysis

To investigate differences between “Pre” vs. “Post” damage groups and for classification of “Mechanical” vs. “Enzymatic” damage groups, multivariate classification analysis based on partial least squares discriminant analysis (PLS-DA) was employed using classification toolbox (version 5.3)^7^ in MATLAB (ver. R2018a, MathWorks, Natick, MA, USA). PLS-DA models were developed using a training set consisting of 85% of the data, and tested on the remaining 15%. Prior to analysis, the joints were split by animals, training and test set were cycled through ten iterations with each joint used once as a test group to avoid biased estimators. Furthermore, leave-one-out cross-validation method was used to estimate classification accuracy for model selection, and a maximum of 10 PLS components were tested.

The partial least squares regression (PLSR) technique was employed in MATLAB to investigate correlation between articular cartilage spectral data of different damage groups and reference measurements of biomechanical properties. PLSR models were validated using test set (15% of data) similar to PLS-DA. Model performance was evaluated using root mean square error of prediction (RMSEP), cross-validation (RMSECV) and the coefficient of determination (*R*^2^) between the actual and predicted values.

### Biomechanical Testing

Biomechanical indentation tests were performed after sample degradation and NIR measurements. The instrument used for biomechanical measurements consisted of a custom made high-precision material testing device (resolution: 0.1 *µ*m, 0.005 N)^21^,^22^,^41^ fitted with a cylindrical indenter (diameter = 0.7 mm). The biomechanical measurements were carried out at the center of specimens, similarly as the NIR measurements. The bone end of the osteochondral samples was glued to the bottom of the measurement chamber, which was then filled with PBS. Perpendicularity between the cartilage surface and indenter tip was adjusted using a goniometer. Control of the measurement and data acquisition were carried out using a custom-made software (LabView, National Instruments). The samples were tested using stress-relaxation protocol for determination of equilibrium modulus (*E*_eq_). A three-step testing protocol (5% of remaining cartilage thickness at each step with 100%/s ramp rate) was applied with the relaxation time between each step being 900 s. Before mechanical indentation, the samples were preconditioned using a cyclic 2% strain (4 full cycles). The stress–relaxation protocol was followed by measurement of the dynamic modulus (*E*_dyn_), for which a sinusoidal dynamic test was performed with frequencies of 0.1, 0.5, 1, and 2 Hz (strain amplitude: 2% of remaining thickness, 4 cycles). The values of equilibrium and dynamic moduli were calculated by assuming the cartilage to be a mechanically elastic and isotropic material.^18^

### Univariate Statistical Analysis

Statistical analysis for the Equilibrium and Dynamic moduli were performed on GraphPad Prism statistical software (version 5.0, GraphPad Software Inc., La Jolla, CA). The data was expressed as mean (Table 1) and compared using one-way ANOVA. A *p* value of less than 0.05 was considered to indicate statistical significance.

**Table 1:** Mean, range and standard deviation (SD) of the biomechanical parameter values.

	Equilibrium modulus (MPa)			Dynamic modulus (MPa)		
	Mean	Range	SD	Mean	Range	SD
Control	1.10	0.33–1.95	0.47	6.55	1.40–12.23	3.18
Enzymatic damage	0.64	0.17–1.37	0.31	4.03	0.72–7.69	1.90
Trypsin, 30 min	0.64	0.20–1.37	0.30	4.82	3.40–6.78	1.31
Collagenase, 90 min	0.72	0.17–1.20	0.31	4.23	2.92–5.66	0.87
Collagenase, 24 h	0.28	0.17–0.44	0.09	3.03	0.72–7.69	2.61
Mechanical damage	0.65	0.21–1.59	0.37	4.56	0.72–7.69	2.46
Impact	0.35	0.21–0.59	0.31	3.04	1.24–5.74	1.33
Abrasion	0.95	0.46–1.59	0.36	6.08	2.86–10.72	2.42

## Results

**Figure 2:**
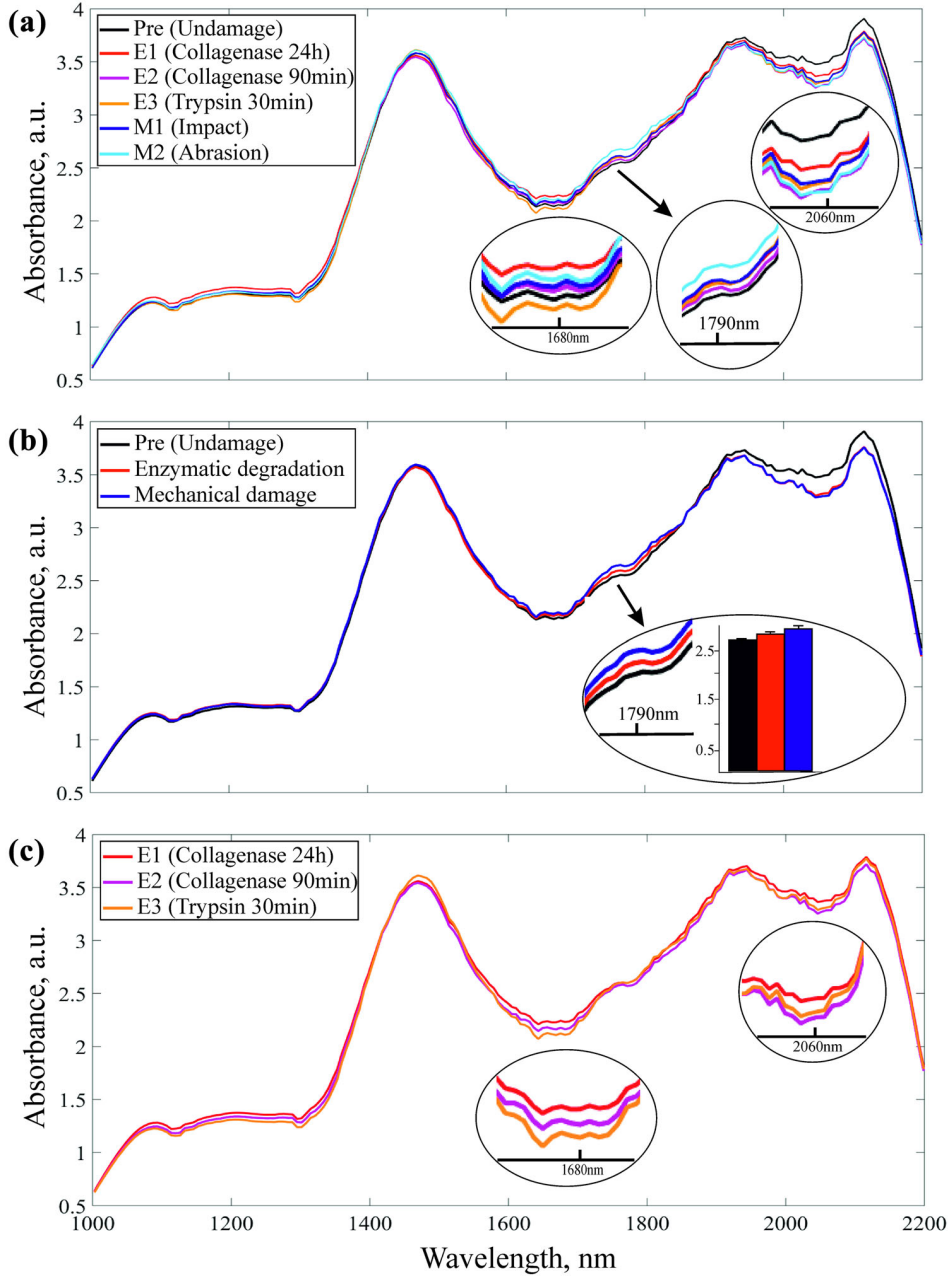
Mean NIR spectra for (a) all groups, (b) mechanical, enzymatic and undamaged (‘Pre’) groups, and (c) enzymatic damages (E1, E2 and E3). Insert figures show major differences between NIR spectra in the region of interest and the bars show standard deviations of mean spectra of 1790 nm wavelength. Colors of the bars correspond to the colors of mean spectra.

Mean NIR spectra of enzymatic and mechanical damage groups, with respective control group, are presented in Fig. 2. The major differences between spectra of intact and degenerated cartilage are observed in the region of 1700–2200 nm. However, substantial spectral differences between enzymatically and mechanically damaged samples can be observed only in the region of 1780–1810 nm, while spectral differences were observed across all groups (Fig. 2a). Figure 2c shows major differences between the 3 enzymatic degradation groups. We did not observe in mean spectra differences in water content (1450 nm band),^26^ while significant variation of water content was observed between control and damaged cartilage (*p* < 0.05) after data pre-processing.

**Figure 3:**
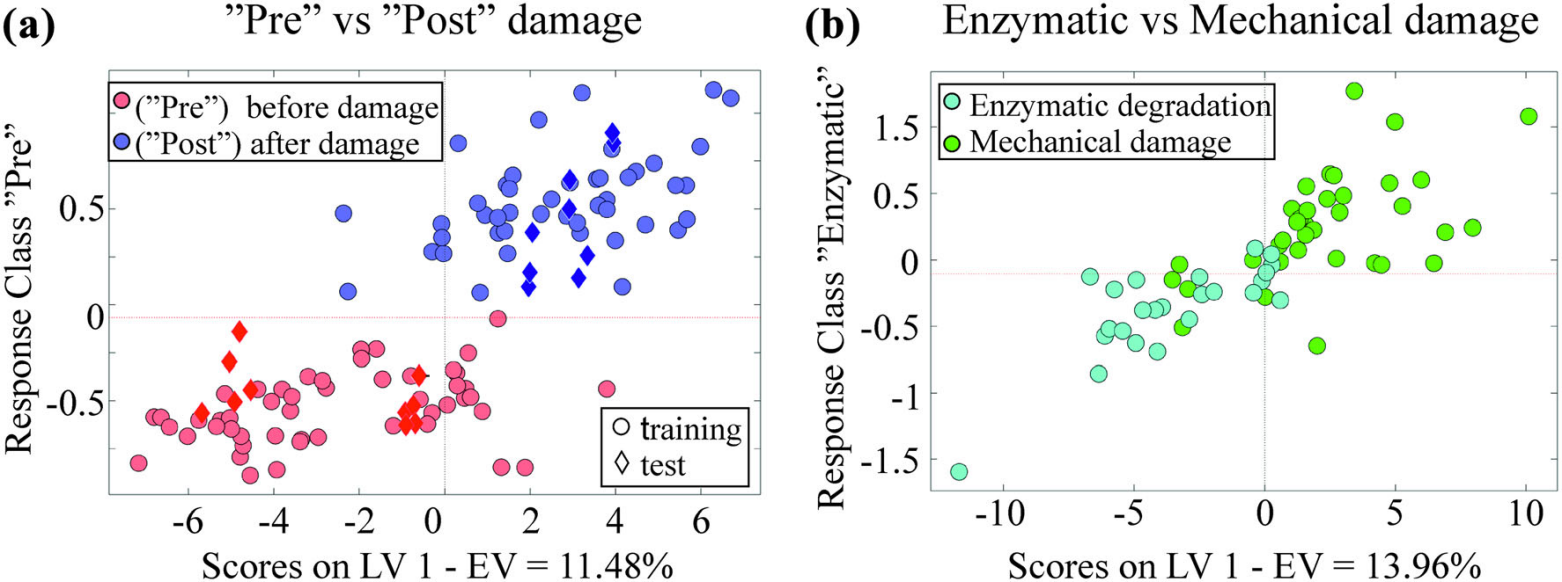
Scatter plot for PLS-DA analysis (a) pre vs. post damage and (b) enzymatic vs. mechanical damage.

The results of two PLS-DA classification models are presented in Fig. 3. The models were created by using 4 PLS components. The first model classifies the samples into two classes: “Pre” (before) vs. “Post” (after) damage (Fig. 3a), while the second model classifies the samples into ‘enzymatic’ vs. ‘mechanical’ damage classes (Fig. 3b). Classification results are presented in Table 2.

**Table 2:** Classification result of PLS-DA analysis.

	Calibration (%)			CV (leave-one-out) (%)			Test (%)		
	Accuracy	Sensitivity	Specificity	Accuracy	Sensitivity	Specificity	Accuracy	Specificity	Sensitivity
“Pre” vs. “post”	98	98	98	93	91	95	86	87	89
“Enzymatic” vs. “mechanical”	97	98	98	85	86	88			

Training set (85% of samples) was used for creating the first model (“Pre” vs. “Post”), and the model was validated using an independent test set (15% of samples). A confusion matrix, showing the model performance on the test set is presented in Table 3. A classification accuracy of 86% was observed for the first model. For the second model (enzymatic vs. mechanical), only leave-one-out cross-validation approach was adopted due to the smaller sample size. A classification accuracy of 83% was observed for this model (Table 4). The misclassified samples observed in the different injury groups are highlighted in the confusion matrix presented in Table 5.

**Table 3:** Confusion matrix of test set for PLS-DA analysis.

	“Pre” damage (%)	“Post” damage (%)
“Pre” damage (%)	87	13
“Post” damage (%)	11	88

**Table 4:** Confusion matrix of leave-one-out cross validation for PLS-DA analysis

	Enzymatic damage (%)	Mechanical damage (%)
Enzymatic damage (%)	86	16
Mechanical damage (%)	13	79

**Table 5:** Confusion matrix of leave-one-out cross validation for PLS-DA analysis of individual injuries groups.

	E1 (%)	E2 (%)	E3 (%)	M1 (%)	M2 (%)	Samples not classified (%)
E1 (%)	75	8	0	0	0	17
E2 (%)	0	43	0	1	1	25
E3 (%)	0	0	91	0	0	9
M1 (%)	0	0	0	50	0	50
M2 (%)	0	0	0	0	66	34

**Figure 4:**
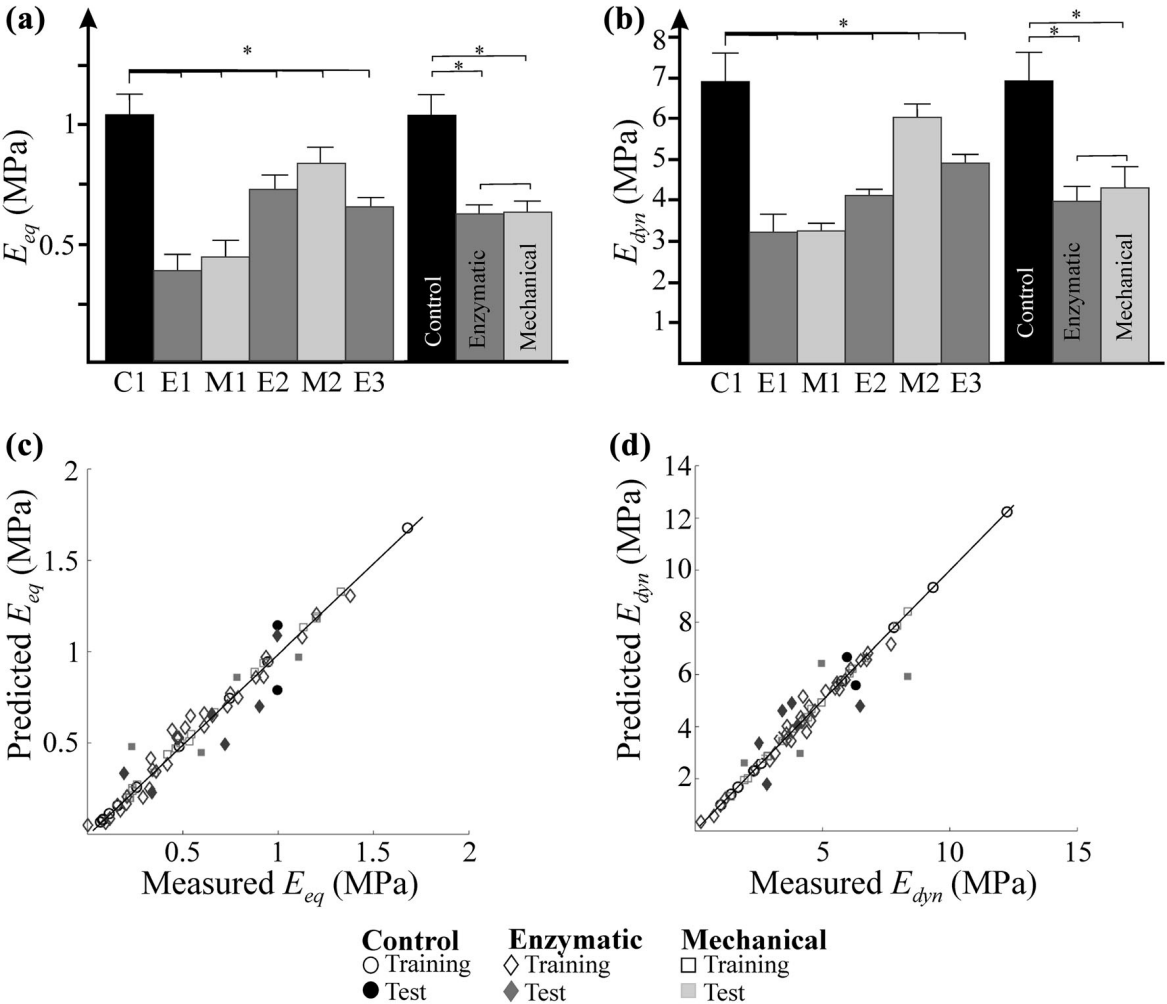
Distribution of (a) equilibrium moduli and (b) dynamic moduli among damage groups and control group. The relationship between NIR spectral measured and predicted (c) equilibrium moduli and (d) dynamic moduli (*p < 0.05).

Significant variation in cartilage biomechanical properties was observed between control and damaged cartilage (*p *< 0.05) (Figs. 4a and 4b). Nevertheless, no significant differences (*p *= 0.184) were observed between the enzymatically and mechanically damaged samples.

PLSR was used to develop models for estimating the biomechanical properties (equilibrium and dynamic moduli) of the samples from their NIR spectral data.^28^ The models were created by using 4 PLS factors. The PLSR models were validated using an independent test set (15% of samples) (Figs. 4c and 4d). High correlations (*R*^2^= 78–95%) were obtained with the optimal models for prediction of the biomechanical parameters (Table 6).

**Table 6:** PLSR model performance for estimating cartilage biomechanical properties for control and different cartilage injury groups.

	Control			Enzymatic damage			Mechanical damage		
	*R*^2^ (%)	RMSEP (%)	RMSECV (%)	*R*^2^ (%)	RMSEP (%)	RMSECV (%)	*R*^2^ (%)	RMSEP (%)	RMSECV (%)
Equilibrium	94	31	26	79	25	12	78	40	12
Dynamic	95	17	18	78	20	8	86	13	8

## Discussion

In the present study, we investigated for the first time the ability of NIR spectroscopy, conducted using a custom fiber-optic arthroscopic probe, to detect differences between enzymatically and mechanically induced damages of articular cartilage *in vitro*. The experimentally induced damages, mechanical and enzymatic injuries, are associated with trauma related structural injury and osteoarthritis related changes in tissue composition, respectively. The findings presented here could prove useful in detecting compromised cartilage prior to visible signs of matrix structural and compositional degeneration.

Classification based on PLS-DA provides an insight into the relationship between the optical response of articular cartilage and the state of its matrix. The high PLS-DA classification accuracy for “Pre” vs. “Post” group classification (cross-validation 93% and test set 86%) suggests significant changes in the tissue structure and/or composition following degeneration. This indicates that NIR spectroscopy is sensitive to overall degenerative changes in the cartilage matrix.

It is a well-known fact that enzymes modify the chemical bonds of molecules,^36^ whereas mechanical damage induces structural changes to the tissue matrix.^15^ Based on these facts, we collected the spectra from abrasion and impact damage groups into “mechanical” damage group and the spectra from trypsin and collagenase damaged samples into “enzymatic” damage group. Compared to differentiating control and damaged tissues, the classification accuracy was lower when differentiating between “enzymatic” degradation and “mechanical” damage groups (cross-validation 85%). Nevertheless, the accuracy indicates that mechanical and enzymatic degradation of articular cartilage can be sensitively distinguished using NIR spectroscopy. The results show poor classification accuracy (48%) in a model that distinguishes between three groups (‘pre’, mechanical and enzymatic damage). Most misclassifications in this model are within the damage groups, this is likely because of the substantial difference between the ‘pre’ vs ‘post’ (damage) classes, and small differences within the damage group. With respect to *in vivo* adaptation, a two-model approach would be required; in the first stage a model that distinguishes between healthy and damaged cartilage will be evaluated, while the second stage model (if the tissue is classified as damaged) will determine the nature of damage (mechanical or enzymatic).

The depth-dependent interactions of NIR light with articular cartilage are related to chemical bonds of the matrix constituents, i.e., water, PG, and collagen, particularly molecules containing OH, CH, NH, and SH bonds.^25^,^36^ Absorption bands in the NIR spectral range are based on overtone and combination vibrations. Changes in the absorbance bands that characterize the matrix components of articular cartilage have been shown to correspond with changes in the matrix structure and composition.^2^,^3^,^5^,^10^,^23^,^39^ Superficial proteoglycan loss is one of the earliest indicators of cartilage degeneration. Since proteoglycans are negatively charged macromolecules that interact with water and swell within the collagen network, depletion of this matrix component results in reduced matrix stiffness and increased water content. This can be observed in changes in the spectra of the damaged groups relative to the control group (Fig. 2), and this can be attributed to an increase in matrix water content. Major differences between the NIR spectra of the different injury groups (Fig. 2) in the region of 1700–1900 nm are due to first C–H overtone bond vibrations, associated with the solid matrix components of the tissue (proteoglycans and collagen). Another noticeable difference in the NIR spectra can be observed in the spectral range of 1900–2200 nm, which is dominated by the absorption band of water (bound and free) at 1924 nm,^26^ with smaller absorbances from N–H bond vibrations. This region also includes the combination band, which makes it difficult to resolve the contribution of the specific matrix components of cartilage to the resulting spectrum.

Biomechanical testing showed a significant decrease equilibrium and dynamic moduli of the enzymatically and mechanically damaged cartilage samples when compared to the control group. The lower values of equilibrium and dynamic moduli in all damaged groups can be attributed to significant loss of the superficial proteoglycans.^22^ The loss of proteoglycans decreases the capacity of articular cartilage matrix to effectively resist compressive loads. In contrast with the results of PLS-DA analysis, mechanical testing did not show significant differences between enzymatically and mechanically induced damage (Fig. 4b). However, we observed large internal variation of equilibrium and dynamic moduli in mechanical damage groups, for example the mean equilibrium modulus for impact and abrasion were 0.35 MPa and 0.95 MPa, respectively. This is due to the different nature of mechanical damages. In the case of surface abrasion, only the superficial layer of cartilage is damaged while impact damage disturbs the collagen network, affecting all the layers of the cartilage. In addition, the biomechanical test was restricted to compression only, which does not necessarily represent the full range of biomechanical response of cartilage. Comparative observations of biomechanics and NIR data suggests that NIR spectroscopy is more sensitive to molecular-level changes in the matrix and is capable of differentiating between structural and biochemical related changes in the tissue matrix,^3^,^5^,^8^,^32^ which is not feasible with biomechanical testing, which only reveals macroscopic changes. Furthermore, the samples were not subjected to physiological loading post-injury, which would have further revealed the true nature and effect of injuries.

The capacity of NIR arthroscopy to distinguish between different cartilage injury types that mimic PTOA and idiopathic OA via mechanical and enzymatic damages, respectively, was evaluated in this study. We conclude that NIR spectroscopy, based on specific spectral features and combined with multivariate classification analysis, has the potential to distinguish between idiopathic and trauma-related degeneration of cartilage, as well as structural injuries with and without PG or collagen losses. This information could be useful in determining the optimal treatment strategy during arthroscopic cartilage repair.

## References

[1] Afara IO, Hauta-Kasari M, Jurvelin JS, Oloyede A, Töyräs J (2015). Optical absorption spectra of human articular cartilage correlate with biomechanical properties, histological score and biochemical composition. Physiol. Meas..

[2] Afara IO, Moody H, Singh S, Prasadam I, Oloyede A (2015). Spatial mapping of proteoglycan content in articular cartilage using near-infrared (NIR) spectroscopy. Biomed. Opt. Express.

[3] Afara I, Prasadam I, Crawford R, Xiao Y, Oloyede A (2012). Non-destructive evaluation of articular cartilage defects using near-infrared (NIR) spectroscopy in osteoarthritic rat models and its direct relation to Mankin score. Osteoarthr. Cartil..

[4] Afara IO, Singh S, Moody H, Zhang L, Oloyede A (2017). Characterization of articular cartilage recovery and its correlation with optical response in the near-infrared spectral range. Cartilage.

[5] Afara I, Singh S, Oloyede A (2013). Application of near infrared (NIR) spectroscopy for determining the thickness of articular cartilage. Med. Eng. Phys..

[6] Anderson DD, Chubinskaya S, Guilak F, Martin JA, Oegema TR, Olson SA, Buckwalter JA (2011). Post-traumatic osteoarthritis: improved understanding and opportunities for early intervention. J. Orthop. Res..

[7] Ballabio D, Consonni V (2013). Classification tools in chemistry. Part 1: linear models. PLS-DA. Anal. Methods.

[8] Baykal D, Irrechukwu O, Lin P-C, Fritton K, Spencer RG, Pleshko N (2010). Nondestructive assessment of engineered cartilage constructs using near-infrared spectroscopy. Appl. Spectrosc..

[9] Borrelli J, Zhu Y, Burns M, Sandell L, Silva MJ (2004). Cartilage tolerates single impact loads of as much as half the joint fracture threshold. Clin. Orthop. Relat. Res..

[10] Brown CP, Bowden JC, Rintoul L, Meder R, Oloyede A, Crawford RW (2009). Diffuse reflectance near infrared spectroscopy can distinguish normal from enzymatically digested cartilage. Phys. Med. Biol..

[11] Brown TD, Johnston RC, Saltzman CL, Marsh JL, Buckwalter JA (2006). Posttraumatic osteoarthritis: a first estimate of incidence, prevalence, and burden of disease. J. Orthop. Trauma.

[12] Buckwalter JA, Mankin HJ (1997). Instructional course lectures, the American Academy of orthopaedic surgeons-articular cartilage. Part II: degeneration and osteoarthrosis, repair, regeneration, and transplantation. JBJS.

[13] Buckwalter JA, Mankin HJ (1998). Articular cartilage: degeneration and osteoarthritis, repair, regeneration, and transplantation. Instr. Course Lect..

[14] Carney SL, Billingham MEJ, Muir H, Sandy JD (1984). Demonstration of increased proteoglycan turnover in cartilage explants from dogs with experimental osteoarthritis. J. Orthop. Res..

[15] Cooke ME, Lawless BM, Jones SW, Grover LM (2018). Matrix degradation in osteoarthritis primes the superficial region of cartilage for mechanical damage. Acta Biomater..

[16] De Vries-van Melle ML, Mandl EW, Kops N, Koevoet WJLM, Verhaar JAN, van Osch GJVM (2011). An osteochondral culture model to study mechanisms involved in articular cartilage repair. Tissue Eng. C.

[17] Harris EDJ, Parker HG, Radin EL, Krane SM (1972). Effects of proteolytic enzymes on structural and mechanical properties of cartilage. Arthritis Rheum..

[18] Hayes WC, Keer LM, Herrmann G, Mockros LF (1972). A mathematical analysis for indentation tests of articular cartilage. J. Biomech..

[19] Hofmann GO, Marticke J, Grossstück R, Hoffmann M, Lange M, Plettenberg HKW, Braunschweig R, Schilling O, Kaden I, Spahn G (2010). Detection and evaluation of initial cartilage pathology in man: a comparison between MRT, arthroscopy and near-infrared spectroscopy (NIR) in their relation to initial knee pain. Pathophysiology.

[20] Horisberger M, Valderrabano V, Hintermann B (2009). Posttraumatic ankle osteoarthritis after ankle-related fractures. J. Orthop. Trauma.

[21] Kokkonen HT, Jurvelin JS, Tiitu V, Töyräs J (2011). Detection of mechanical injury of articular cartilage using contrast enhanced computed tomography. Osteoarthr. Cartil..

[22] Korhonen RK, Laasanen MS, Töyräs J, Rieppo J, Hirvonen J, Helminen HJ, Jurvelin JS (2002). Comparison of the equilibrium response of articular cartilage in unconfined compression, confined compression and indentation. J. Biomech..

[23] Marticke JK, Hösselbarth A, Hoffmeier KL, Marintschev I, Otto S, Lange M, Plettenberg HKW, Spahn G, Hofmann GO (2010). How do visual, spectroscopic and biomechanical changes of cartilage correlate in osteoarthritic knee joints?. Clin. Biomech..

[24] Moody HR, Brown CP, Bowden JC, Crawford RW, McElwain DLS, Oloyede AO (2006). In vitro degradation of articular cartilage: does trypsin treatment produce consistent results?. J. Anat..

[25] Oluwaseun AI, Zenon P, Adekunle O (2011). Current state of the application of infrared optical methods for assessing articular cartilage. J. Mater. Sci. Eng. A.

[26] Padalkar MV, Spencer RG, Pleshko N (2013). Near infrared spectroscopic evaluation of water in hyaline cartilage. Ann. Biomed. Eng..

[27] Prakash M, Joukainen A, Torniainen J, Honkanen MKM, Rieppo L, Afara IO, Kröger H, Töyräs J, Sarin JK (2019). Near-infrared spectroscopy enables quantitative evaluation of human cartilage biomechanical properties during arthroscopy. Osteoarthr. Cartil..

[28] Prakash M, Sarin JK, Rieppo L, Afara IO, Töyräs J (2017). Optimal regression method for near-infrared spectroscopic evaluation of articular cartilage. Appl. Spectrosc..

[29] Prakash M, Sarin JK, Rieppo L, Afara IO, Töyräs J (2017). Optimal regression method for near-infrared spectroscopic evaluation of articular cartilage. Appl. Spectrosc..

[30] Puhakka PH, te Moller NCR, Tanska P, Saarakkala S, Tiitu V, Korhonen RK, Brommer H, Virén T, Jurvelin JS, Töyräs J (2016). Optical coherence tomography enables accurate measurement of equine cartilage thickness for determination of speed of sound. Acta Orthop..

[31] Saarakkala S, Toyras J, Hirvonen J, Laasanen MS, Lappalainen R, Jurvelin JS (2004). Ultrasonic quantitation of superficial degradation of articular cartilage. Ultrasound Med. Biol..

[32] Sarin JK, Nykänen O, Tiitu V, Mancini IAD, Brommer H, Visser J, Malda J, van Weeren PR, Afara IO, Töyräs J (2019). Arthroscopic determination of cartilage proteoglycan content and collagen network structure with near-infrared spectroscopy. Ann. Biomed. Eng..

[33] Sarin JK, te Moller N, Brommer H, van Weeren R, Mancini I, Malda J, Afara IO, Töyräs J (2018). Arthroscopic near infrared spectroscopy enables simultaneous quantitative evaluation of articular cartilage and subchondral bone in vivo. Sci. Rep..

[34] Saukko AEA, Honkanen JTJ, Xu W, Vaananen SP, Jurvelin JS, Lehto V-P, Toyras J (2017). Dual contrast CT method enables diagnostics of cartilage injuries and degeneration using a single CT image. Ann. Biomed. Eng..

[35] Shingleton WD, Hodges DJ, Brick P, Cawston TE (1996). Collagenase: a key enzyme in collagen turnover. Biochem. Cell Biol..

[36] Sophia Fox AJ, Bedi A, Rodeo SA (2009). The basic science of articular cartilage: structure, composition, and function. Sports Health.

[37] Spahn G, Klinger HM, Hofmann GO (2009). How valid is the arthroscopic diagnosis of cartilage lesions? Results of an opinion survey among highly experienced arthroscopic surgeons. Arch. Orthop. Trauma Surg..

[38] Spahn G, Plettenberg H, Nagel H, Kahl E, Klinger HM, Mückley T, Günther M, Hofmann GO, Mollenhauer JA (2008). Evaluation of cartilage defects with near-infrared spectroscopy (NIR): an ex vivo study. Med. Eng. Phys..

[39] Stumpfe ST, Pester JK, Steinert S, Marintschev I, Plettenberg H, Aurich M, Hofmann GO (2013). Is there a correlation between biophotonical, biochemical, histological, and visual changes in the cartilage of osteoarthritic knee-joints?. Muscles. Ligaments Tendons J..

[40] Torniainen, J., I. O. Afara, M. Prakash, J. K. Sarin, L. Stenroth, and J. Töyräs. Automated preprocessing of near infrared spectroscopic data. In: Biophotonics Congress: Optics in the Life Sciences Congress 2019, OSA Technical Digest (Optical Society of America, 2019), paper DS2A.6.

[41] Toyras J, Laasanen MS, Saarakkala S, Lammi MJ, Rieppo J, Kurkijarvi J, Lappalainen R, Jurvelin JS (2003). Speed of sound in normal and degenerated bovine articular cartilage. Ultrasound Med. Biol..

[42] Wagner M, Werner A, Gründer W (1999). Visualization of collagenase-induced cartilage degradation using NMR microscopy. Invest. Radiol..

[43] Wang Q, Zheng Y-P, Qin L, Huang Q-H, Lam W-L, Leung G, Guo X, Lu H-B (2008). Real-time ultrasonic assessment of progressive proteoglycan depletion in articular cartilage. Ultrasound Med. Biol..

